# Understanding fan pressures and its impact on football club sustainability: insights from the Colombian context

**DOI:** 10.3389/fspor.2024.1508164

**Published:** 2024-12-17

**Authors:** Juan Alejandro Hernández-Hernández, Abraham Londoño-Pineda, Jose Alejandro Cano

**Affiliations:** Faculty of Economic and Administrative Sciences, University of Medellin, Medellin, Colombia

**Keywords:** fan types, solidarity pressures, sustainability outcomes, football club, Colombian football

## Abstract

The framework of stakeholder pressures in sports industries, and in the specific case of football, has been used to identify the reasons why management bodies incorporate interested parties into their business strategy. This is primarily contingent on the pressures that interested parties generate. One of the most influential stakeholders is the fan base, given the emotional attachment that this type of sport evokes in them, commonly referred to as “the fan's affective connection.” Despite the existence of studies on diverse typologies of fans, no investigation has been conducted into the impact these have on the sustainability outcomes of football clubs. Moreover, most existing studies have focused on European leagues, with a pronounced emphasis on the environmental dimension of sustainability. Therefore, the aim of this research is to evaluate the impact of diverse fan types on the sustainability performance of Colombian football clubs, with a specific focus on the triple bottom line approach. To this end, two major fan typologies—active and non-active—were defined based on the findings of the literature review, as well as the pressures associated with solidarity-based factors. A conceptual model and an estimation based on the structural equation model related the different fan typologies and their associated pressures to sustainability outcomes. The results indicate that among the solidarity-based pressures, fans valued social commitment the most, followed by the promotion of women's football and the fight against racism. Environmental engagement was the least relevant, but still positively rated, suggesting the need for awareness-raising efforts to extend fans' sustainability practices beyond the stadium. The findings of this research can inform strategies for football clubs to engage fans and improve their sustainability performance across economic, social and environmental dimensions.

## 1 Introduction

The effects of climate change have prompted governments and companies to engage in the formulation of proposals aimed at curbing emissions and other adverse impacts, as outlined in the Paris Climate Change Agreement (1) and the commitments set forth to achieve the Sustainable Development Goals (SDGs) (2, 3). Despite the efforts of different sectors, a significant number of these initiatives have been limited in their implementation due to the lack of binding commitments for organizations (4). The sports industry has not been exempt from these requirements. Both the sporting and academic sectors have reached a consensus that this field plays a pivotal role in driving the transition to a more sustainable global environment (5, 6). The relationship between sport and sustainability is beginning to be recognized as an emerging field of research (7). The explanation can be found in the potential impact of sports organizations on various stakeholders (8–10). However, the sports industry, especially football, has been slow to engage with these issues, either entering the debates late or only recently starting to address issues of corporate social responsibility (CSR) and corporate sustainability (CS) (11).

One factor contributing to this phenomenon is the persistence of the notion that the primary objective of managerial practice is to maximize shareholder profits (12). This explains why firms' business models have historically been oriented toward creating value primarily for their shareholder (13, 14). In contrast to this perspective, stakeholder-based approaches suggest that the conventional short-term goal of maximizing profits for shareholders, which represents the traditional understanding of value creation, should be replaced (15). Consequently, there is a need to move towards the concept of shared value creation (16, 17), which gives rise to the concept of sustainability. This is because value creation would be expressed in terms of expected outcomes in the economic, social and environmental spheres (18). Consequently, a framework for applying stakeholder theories to sport industries has been developed by McCullough and Cunningham (19). They argue that managers are motivated to include stakeholders in their business strategy, primarily because of the pressure they generate. The empirical validation of this framework in the context of European football clubs has been developed in the works of Daddi et al. (20, 21), and Todaro et al. (22). These studies have found evidence that the governing bodies of European football clubs participate in sustainability initiatives and practices motivated by pressure from their stakeholders.

In this regard, it should be noted that the football sector comprises a variety of stakeholders, some of which have been classified as external and internal (9, 23), while others have identified them as primary and secondary stakeholders (13). Furthermore, some have categorized them as institutional stakeholders (18, 21), while others have considered them as market and societal stakeholders (22). These stakeholders exert pressure on the managers of football clubs and lack the characteristics associated with football fans, largely due to the emotional intensity and passion that characterizes this particular sport (24–26). This phenomenon has been referred to as the “the fan's affective connection” (27, 28), which creates a long-term relationship between fans and the club with which they identify (29). These factors ensure a consistent demand for the club's goods and services. Consequently, any failure to acknowledge or address the relationship with fans could have significant financial implications for the club (30–32). In addition, fans have the ability to exert significant pressure on the performance of a club due to the presence of a “sense of urgency” (33–35).

For this reason, this research focuses on the study of fans of football clubs, providing an examination of fan typologies and their multidimensional impact on the sustainability of football organizations. Therefore, the aim of this study is to assess the influence of different fan types on the sustainability performance of professional football clubs. To this end, a survey was conducted in South America among fans of different Colombian football clubs. The selection of this country is based on several factors. The first reason for this choice is that no studies have been identified that relate fan pressures to the sustainability performance of football clubs, either in South America or in Colombia. The studies that have been conducted on this topic, such as the works of Fernández-Villarino (11), Daddi et al. (21), Cayolla et al. (23), Lozano and Barreiro-Gen (36), and Todaro et al. (22), were conducted only in European leagues. Secondly, as in Argentina, Brazil and Uruguay, the culture of football is particularly strong in Colombia (37). Indeed, football plays a significant role in the social, cultural and economic development of numerous communities and population groups, which in turn has an impact on economic growth (38). Thirdly, Colombia has developed a reputation for producing high-quality players who are subsequently exported to other international leagues, including those in Europe (39). This situation resulted in Colombia becoming the fifth largest exporter of players worldwide between 2017 and 2022 and, since 2020, the third largest exporter of players in South America, behind Brazil and Argentina (40). Furthermore, the Colombian men's national football team maintained an undefeated record of 28 games between 2022 and 2024. Additionally, it secured second place in the Copa América 2024 and, as of October 2024, remains in the top 10 teams in the FIFA ranking (41).

It is also important to note that the pressures exerted by the fans have prompted a shift in the business models of Colombian professional football clubs. These changes have primarily involved a reorientation towards alternative revenue streams, including television rights, ticket sales, merchandise, and other commercial opportunities (42). However, recently, there has been a shift in focus towards the social commitment of football clubs. This is evidenced by initiatives such as work with children, vulnerable groups, the adoption of pets, and others that are part of the programs of football institutions such as FIFA. These initiatives include the fight against racism and the promotion of women's football (43). Regarding the potential of women's football, it can be stated that this sport is gaining momentum in Colombia. This country served as the host nation for the U-20 Women's World Cup in September 2024. In two matches, attendance records were surpassed that had previously stood for the entirety of the history of the U-20 Women's World Cups. The first was in the city of Medellín in the match between Colombia and Mexico, with an estimated 35,800 spectators in attendance. The second match was held in the city of Cali between Colombia and the Netherlands, with an estimated 37,300 fans in attendance.

The remainder of the articles is organized as follows: Section 2 presents the theoretical framework that allows for the definition of fan typologies and the characterization of the pressures they generate. Section 3 outlines the methodology, which presents the questionnaire used and the conceptual model that supports the evaluation of the expected effects on the sustainability of football clubs derived from the pressures generated by fans and identifies the expected outcomes of sustainability in the football industry. Section 4 presents the results of the SEM, while the discussion of.

## 2 Literature review and theoretical framework

The study of stakeholders in sport has been approached from the perspective of the framework proposed by McCullough and Cunningham (19), McCullough et al. (9) and McCullough et al. (18), who presented a study framework based on institutional theory. From an institutional perspective, it is more accurate to speak of institutional stakeholders, which include government stakeholders, football institutions, and market and societal stakeholders. This last group also includes fans (20–22). This study focuses on the stakeholder group of fans because they are distinguished from other stakeholders by their unique “affective connection” with the sport (24, 29). This emotional bond fosters a sense of loyalty and commitment to the club, which in turn may influence their long-term patronage of the football club's services (35, 44).

### 2.1 Typologies of fans

While some fan taxonomies have been identified in the football literature to serve as a starting point for analysis, there is still a lack of work that establishes a relationship between the pressures of these typologies and the sustainability outcomes of football clubs (10). The foundational works on fan typologies were developed by Mitchell and Wood (33) and Senaux (34). These authors proposed three criteria for classifying fans that help explain their different levels of identity: legitimacy, power and sense of urgency. The concept of legitimacy is related to aspects such as the trust that fans have in the football club, which leads to loyalty and long-term commitment (22, 44, 45).

The power of fans depends on their ability to influence the financial decisions of football clubs, both in the short and long term. This is achieved through a variety of means, including attendance at matches, the purchase of broadcast rights and the acquisition of club-related merchandise (27, 32). Conversely, a sense of urgency is the most salient characteristic of fans who are truly committed to their football club. When the club's performance is suboptimal, they demand appropriate reinforcement or perceive that managers are not providing it. In the absence of a genuine sporting project, these individuals are the most vocal in their demands (26, 46, 47).

Table 1 identifies four typologies of fans: direct, indirect, passive and potential. In the case of direct fans, it must be recognized that these are those who directly follow the football club with which they typically identify. This is usually achieved either by attending matches and purchasing season or individual tickets (51, 52). This type of fan is perceived as having high legitimacy due to their demonstrated loyalty and trust in the football club (11, 24, 29). Likewise, they have considerable influence as their commitment is essential for the long-term financial stability of the football club (22, 35, 53). In addition, there is a heightened sense of urgency as any adverse events that affect the club's performance are perceived as personal, including losses in important games, finals, or unfavorable outcomes (26, 45).

**Table 1:** Characteristics of the different types of fans.

Authors	Direct Fans	Indirect Fans	Passive fans	Potential fans
Mitchell and Wood (33); Senaux, (34)	High legitimacy, high power, high sense of urgency	High legitimacy, high power, high sense of urgency	Low legitimacy and power and low sense of urgency	Low legitimacy and power and low sense of urgency
Giulianotti (48)	High loyalty and solidarity with the soccer club	High loyalty and solidarity with the soccer club	Low legitimacy and power and low sense of urgency	Low loyalty and solidarity with the soccer club
Tapp (49)	High loyalty	High loyalty	Low loyalty	Low loyalty
Kellison and Kim, (50)	High Identification and loyalty	High Identification and loyalty	Low identification and loyalty	Low identification and loyalty
Parganas (35)	High Participation	High Participation	Low involvement	Low involvement
Jaeger (26)	Active fans: high identification and loyalty	Active fans: high identification and loyalty	Passive fans: low identification and loyalty	Passive fans: low identification and loyalty
Winskowski (29)	Direct fans: they attend the stadium directly	Indirect fans follow the matches through different means of transmission: digital, satellite, online, etc.	Not available	Not available
Cayolla et al. (23)	Active fans: high identification and loyalty	Active fans: high identification and loyalty	Passive fans: low identification	Green consumers

Conversely, indirect fans exhibit a heightened sense of urgency due to their identification with the club. This generates a high level of legitimacy, as sporting results do not affect their loyalty to the clubs they support. This type of fan typically follows the club via television or the Internet, through a subscription to the operators that broadcast the matches (48, 54). This behavior gives the fan considerable influence over the club's finances (26). In contrast, passive fans may follow the club intermittently through various broadcast media. However, they tend to have a low sense of urgency, which ultimately leads to a reduced sense of legitimacy due to their limited identification with the football club. As a result, they have a minimal influence over the financial aspects of the club. A review of the literature shows that this classification is similar to that proposed by Giulianotti (48) and Jaeger (26), who referred to these individuals as “fans” and “followers,” respectively.

It is also possible to identify a category of potential fans who do not currently support any football club. This group may not feel a sense of urgency, legitimacy, or power in following or demanding the services of a club. However, they may become emotionally attached to a club if the club offers something that appeals to them. This phenomenon has been termed “green consumers” by Cayolla et al. (23). These types of fans have a pro-environmental mentality and thus demonstrate a certain willingness to participate in sports initiatives that include sustainability components (20, 55). Among the activities related to sustainability components that clubs can implement are those of a social nature, such as community engagement (56), approaches to gender perspectives and the promotion of women's football (57, 58), and the orientation towards the development of projects that promote environmental awareness among fans (21, 22).

### 2.2 Types of pressure generated by fans

Fan behavior can influence a number of groups involved in sport, including referees (59), coaches (60), players (61), and visiting clubs (62). However, in this study, the focus of pressure shifts to sports organizations (63). This type of pressure is a consequence of the unique nature of football and the deep attachment that fans have to the club they support (23). In order to address the type of pressure that fans exert on the football industry, institutional theory is required as it explains how organizations, including football organizations, respond to pressure from different stakeholders, which represent the expectations and demands to be met (26, 45). In this context, the concept of isomorphism is key to understanding the pressures that fans place on football organizations. Isomorphism can be defined as the standardized or homogenized behavior of firms that results from social norms and behaviors (19). Accordingly, DiMaggio and Powell (64) propose three types of pressure that shape the conceptualization of isomorphism: coercive, mimetic and normative.

Coercive pressures are defined as those that are mandatory or, in the absence of a requirement, those that become common practice within an industry or society, thereby influencing firms to conform to this type of demand (65). In this regard, McCullough et al. (9) note that they are most prevalent in the early stages of environmental sustainability practices. These types of pressures mainly originate from government stakeholders and football bodies, given their significant influence on legislative and regulatory matters (21, 22, 66). Mimetic pressures, on the other hand, are more closely associated with the football industry, as they tend to emulate the actions of their counterparts (18, 67). As a result, they have typically emerged during the second wave of sustainability practices, as outlined by McCullough et al. (9). Likewise, there are normative pressures, which are more closely associated with the dissemination efforts of educational institutions (5, 8), and refer to customs, traditions, and social behaviors (68). In the context of football, traditions and customs have a significant influence on the behavior of a range of stakeholders, including football institutions, government institutions, fans and society at large (43).

Additionally, de Witte and Zglinski (69) provide a more detailed typology of fan pressure, which can come from three sources: identity commitment, sporting merit and solidarity. The concept of identity commitment has been defined in different ways. Nevertheless, the majority of the existing literature has linked this phenomenon to the local traditions of the city or region (70). Indeed, for some, football constitutes a symbolic space for the construction of regional identities (71). The commitment to identity is also linked to the processes that contribute to the reduction of information asymmetries and better communication with the public of interest, including fans, since much of the management is focused on building fan loyalty (46). Consequently, the business model should be oriented towards the positive management of fans' emotions (24). Some scholars have suggested that managers should be fans of the clubs they manage. Others have even suggested that, in order to improve management, we should move towards a model where fans are also owners of clubs (72). The emphasis on sporting achievement suggests that fans attach importance to their clubs winning local and international tournaments. Consequently, there is a need for clubs to focus on achieving sporting success. Moreover, the emotional bond between fans and their clubs leads to a sense of personal responsibility for the club's performance, including their defeats (45, 53). One way to achieve sporting merit is for managers to acquire skilled player (26) and even a competent coaching staff, as coaches serve as the public face of the club and are the focus of fans' positive and negative feelings about the club's performance (73).

The pressure associated with solidarity is linked to selected initiatives of the SDGs agenda (57) and is generated because fans positively value the fact that the club they support supports social programs (32). Among these programs, work with children's foundations and support for training centers stand out (74). In addition, the variables that make up the pressure associated with solidarity, shown in Table 2, are also linked to the elimination of discriminatory practices. This is illustrated by international campaigns against discrimination, such as the “Not to Racism” initiative (82). Furthermore, there is a correlation between these pressures and the support of current SDG initiatives, including gender equality and the promotion of women's football (57), strategies and actions promoting the protection of the environment, and the dissemination of environmental awareness among stakeholders (22). It is worth noting that, of the potential fans mentioned in Table 1, the “green consumers” identified by Fernández-Villarino (11) and Cayolla et al. (23) may be more likely to experience solidarity pressures stemming from environmental practices and awareness.

**Table 2:** Variables associated with solidarity-based pressures.

Variables	Authors
Social programs	Castillo (75); Wann and James (32); Hernández-Hernández et al. (43); D’auria et al. (74)
Promotion and potentization of women's soccer	Pope and Kirk (76); Valenti et al. (57); Clarkson and Philippou (58); Maguire (77)
Fight against racism and discrimination	Lusted (78); Paramio Salcines et al. (79); Ahn and Cunningham (80); Krech (81)
Environmental practices in the stadium	Daddi et al. (20, 21); Cayolla et al. (23); Todaro et al. (22)
Promotion of environmental awareness among fans	Trendafilova and Mccullough (5); Daddi et al. (20, 21); Fernández-Villarino (11); Cayolla et al. (23); Lozano and Barreiro-Gen, (36)

### 2.3 Sustainability outcomes in the football industry

The football industry is no stranger to international debates and movements on environmental issues and sustainability. However, despite its social and economic importance, it has been slower than other sectors to integrate sustainability principles into its management (11, 36, 83). This is because the issue is not yet binding or mandatory. As a result, most football organizations engage in these initiatives driven by cost savings and economic incentives. In some cases, such initiatives are undertaken with the aim of increasing the positive perceptions and goodwill that fans have towards the organization (84). However, the present study postulates that the main reason for the more definitive forays of football clubs or clubs into sustainability practices is the influence of stakeholders (20), especially fans, as evidenced in the present study.

In this context, sustainability represents a higher purpose, namely the creation of value that goes beyond mere monetary profit (16). Rather, it begins to be expressed as a triple bottom line, which according to Elkington (85) reflects expected outcomes in the economic, social and environmental spheres (14, 86). However, a review of the literature revealed that many studies have focused solely on the economic dimension of sustainability, as this is essential for the long-term survival of the football club or club. Other researchers were tasked with examining the environmental dimension of sustainability, focusing on pro-environmental actions (21), particularly in stadiums and other venues where large numbers of people attend sporting events. This is because *in situ* sustainability is closely linked to the logistical operations of such events (87).

The term 'shared value' has been attributed to various authors, but the essence of the concept transcends traditional shareholder or owner-based value creation (16). This evolution in thinking has led to a shift in focus from a short-term, shareholder-centric approach to a long-term, sustainable one (88). This transition has implications for the business model (89), with sustainability expectations influencing the desired outcomes (84). This, in turn, requires a triple balance between economic, social, and environmental factors (14). In the case of studies that has focused on the relationship between sport industries and sustainability, McCullough and Cunningham (19) can be taken as a starting point for presenting four types of expected outcomes of sustainability, derived from the functional, social and political pressures exerted by fans. These include cost savings, competitive advantage, perceptions of the goodwill of the football club and fan identification. When these outcomes are grouped according to the dimensions of sustainability, it can be argued that cost savings and competitive advantage fall within the economic dimension, whereas football club goodwill and fan identification fall within the social dimension. The above work is one of the pioneering studies in the field, but it did not consider the environmental dimension in its performance results.

In the context of the football industry, the work of Daddi et al. (21) proposes a model in which a number of mimetic, normative and coercive pressures have an impact on environmental performance outcomes in terms of governance practices, environmental practices and environmental operational practices. It is noteworthy that the work in question includes the environmental aspect of sustainability, but does not consider the other dimensions, as the primary focus was on the environmental domain. In contrast, Todaro et al. (22) present a model that considers the three dimensions of sustainability and incorporates the influence of different stakeholders, including government stakeholders, football institutions and market and societal stakeholders such as local communities, fans and sponsors. The pressures exerted by these different actors affect performance outcomes, which are expressed in four expected benefits: internal management, environmental performance, reputation and goodwill, and business and sponsorship. Cayolla et al. (23) state that fan pressure influences a number of performance outcomes, which they refer to as social, environmental and economic benefits. This work is the most like the one presented in this paper as it addresses the same audience (fans) and covers the three dimensions of sustainability.

Consequently, the present study is based on the triple bottom line approach, encompassing economic, social, and environmental considerations (14). About the economic dimension, it has been established that fans can generate income for football clubs in several ways. These include the purchase of individual or season tickets (51, 52), the purchase of club merchandise or services such as souvenirs and sportswear (31, 32, 35), and through broadcast revenue when they subscribe to a sports channel (26, 34, 53). In terms of the social dimension, the most common variable is reputation and goodwill (20–22). On the other hand, within the environmental dimension, environmental practices and dissemination of environmental awareness among fans are identified as the most prominent (10, 23, 36). Table 3 illustrates the expected sustainability outcomes in the football industry and their associated variables.

**Table 3:** Expected sustainability outcomes in football clubs.

Sustainability outcomes	Specific outcomes	Authors
Economic outcomes	Individual and seasons tickets	Cleland and Dixon (51); Cho and Lee (30); Fry et al. (46); Jaeger (26); Leitner and Richlan (52); Ferraresi and Gucciardi (47)
	Sales of goods and services	Cho and Lee (30); Mastromartino et al. (27, 28); Moital et al. (31); Wann and James (32)
	Income from broadcast rights	Senaux (34); Mastromartino et al. (53); Jaeger (26)
Social outcomes	Reputation and goodwill	Daddi et al. (21, 67); McCullough et al. (18); Cayolla et al. (23); Todaro et al. (22)
Environmental outcomes	Outcomes of environmental practices in the stadium	Daddi et al. (21); Cayolla et al. (23); Todaro et al. (22)
	Environmental awareness dissemination outcomes	Daddi et al. (20, 21); Fernández-Villarino (11); Cayolla et al. (23); Lozano and Barreiro-Gen (36)

The conceptual elements of this study therefore focus on fan typologies, solidarity related pressures and expected sustainability outcomes. Their integration constitutes the conceptual model underpinning the measurement of the impact of the pressures of fans on the performance of football clubs, which is discussed in the methodology section.

### 2.4 Conceptual model of solidarity-based pressures and sustainability outcomes

The pressures associated with sporting identity and merit are primarily focused on the sporting performance of the club (69). To achieve this, football clubs and clubs must have players and coaching staff capable of performing at the highest level (90). Moreover, studies have shown a correlation between sports performance indicators and financial performance indicators of clubs (91). Consequently, the outcome of sporting events can also have a significant impact on the valuation of a football club (92). However, the fans who have a more direct impact on club finances are the active fans, i.e., those who attend the stadium through individual or season tickets (51), such individuals purchase goods and services from football clubs, or pay for subscriptions to sports channels (27, 28, 53). However, the evidence did not support the hypothesis that an active fan (direct or indirect) would be more supportive of the club, or that non-active fans (passive and potential) would become new fans or more committed to a football club due to the pressures associated with solidarity (43). Given that these are fan evaluations or assessments, this does not necessarily mean that these are actual revenues for the football clubs, so Hypothesis 1 would be as follows:

**H1:** The solidarity-based pressure exerted by fans has a significant impact on the economic performance of football clubs.

Similarly, football clubs that demonstrate greater social commitment, such as such as support for children's foundations and training centers (74), initiatives to promote women's football (57), the implementation of anti-discrimination and anti-racism programs (82), strategies to promote environmental practices in the stadium and environmental awareness among fans (10, 36), have been shown to have a positive impact on the reputation and goodwill of football clubs (43). In addition, goodwill has been identified as a key expected outcome in conceptual models developed by scholars examining the pressures exerted on football clubs by various stakeholders. These include models proposed by McCullough et al. (18), Daddi et al. (21), Cayolla et al. (10), and Todaro et al. (22). Given the evidence, this study proposes the following hypothesis:

**H2:** The solidarity-based pressure exerted by fans has a significant impact on the social performance of football clubs.

The solidarity pressure associated with environmental commitment facilitates the implementation of environmental practices within the stadium and contributes to the promotion of environmental awareness among fans (11, 23). In this context, the third hypothesis can be formulated as follows:

**H3:** The solidarity-based pressure exerted by fans has a significant impact on the environmental performance of football clubs.

**Figure 1:**
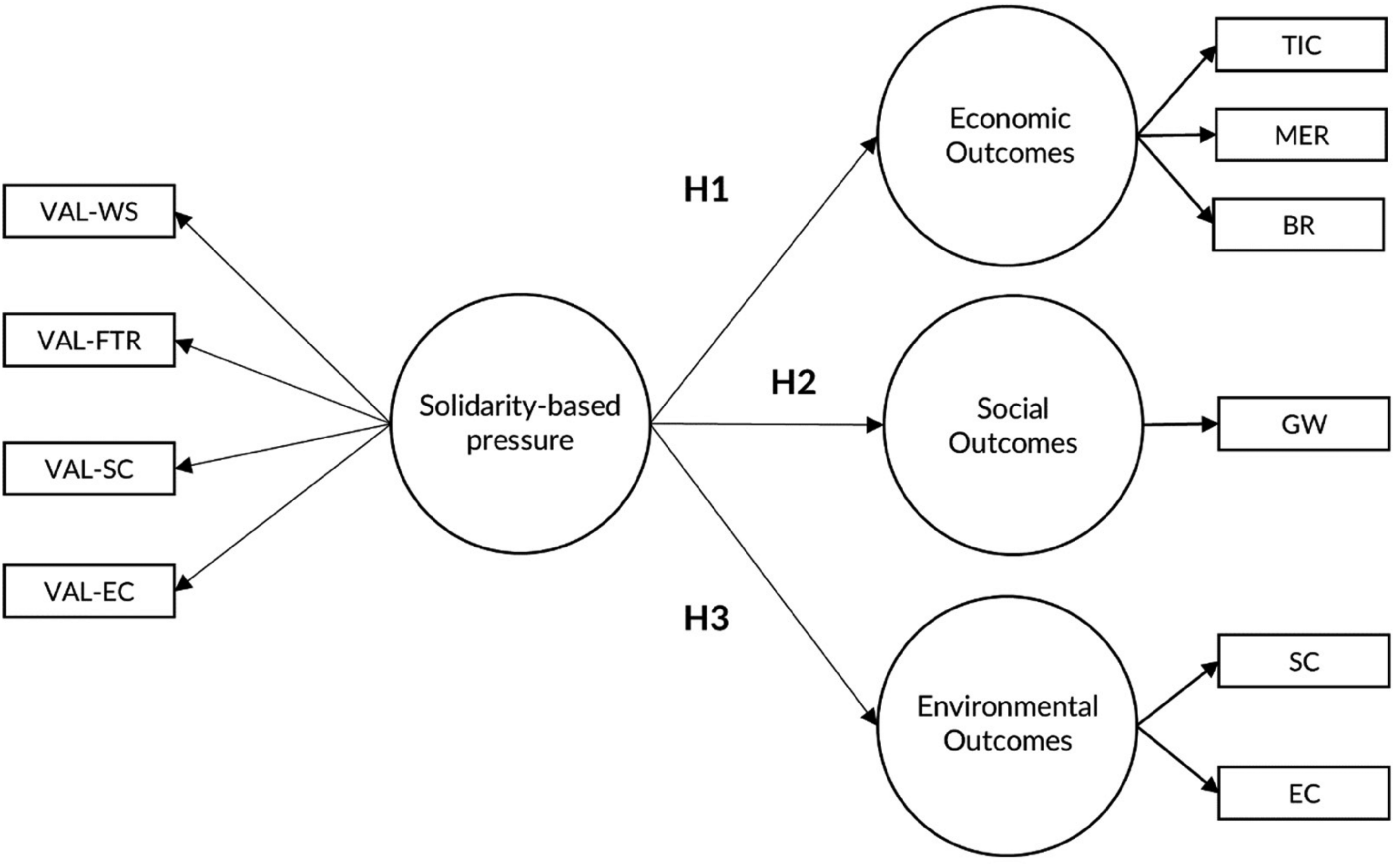
Conceptual model of solidarity-based pressures and sustainability outcomes.

Table A1 from Appendix A presents the variables that make up the conceptual solidarity pressure exerted by fans and the sustainability outcomes of football clubs, while Figure 1 presents the conceptual model that underpins the hypotheses formulated. In this model, solidarity pressure represents a construct, or first-order latent, made up of several observed variables, such as support for women's football, the fight against discrimination and racism, social commitment and environmental commitment. The expected outcomes of sustainability, covering the economic, social and environmental dimensions, in turn constitute the second-order latent variables. These are further constituted by several observable variables, including income from individual and season tickets, income from merchandising, income from broadcasting rights, reputation and goodwill, environmental practices in the stadium and the development of environmental awareness among fans.

## 3 Methodology

### 3.1 Questionnaire design

In order to investigate the influence of fan pressure on the long-term viability of football clubs in Colombia, a questionnaire shown in Appendix B (Tables B1–B7) about perception on fan solidarity pressure and its impact on the sustainability of football clubs was designed.

In the first part of the questionnaire, respondents were informed that their data would not be published as the purpose of this survey was purely academic. The questionnaire included a general section in which respondents were asked to provide information on their first name, surname, place of residence, age group and gender. A taxonomic classification of the profiles was then made based on a question designed to categorize the respondents according to the typology of fans identified, including four categories of fans (direct fans, indirect fans, passive fans, potential fans) and two different groups of fans, called active and non-active fans. Active fans were classified as direct and indirect (29), while non-active fans were classified as passive (43) and potential (23). To do this, respondents were asked to indicate which of the statements in Table 4 best describes their status as a fan.

**Table 4:** Fan status options for respondents.

Fan status	Fan type	Fan group
You visit the stadium on a regular basis with an individual or season ticket.	Direct fan	Active fan
You go to the stadium sporadically (for important matches or finals) or follow your football club regularly via a TV channel that broadcasts the matches (ESPN, Sky Sports, GO Sports, Eurosport, Win Sport).	Indirect fan	
You support a football club, you're happy when they do well, but you don't go to the stadium or follow the club via a TV channel that broadcasts the matches.	Passive fan	Non-active fan
You are not a fan of a particular club, but if a football club is involved in activities that promote women's football, environmental and social practices, you might consider supporting them directly or indirectly.	Potential fan	

In the second part of the questionnaire, respondents are asked about solidarity pressures and their relationship to the dimensions of sustainability, in order to establish their appreciation and importance of solidarity pressures such as support for women's football, the fight against racism and discrimination, social commitment and environmental commitment, and the incidence of these pressures on sustainable outcomes related to the economic, social and environmental dimensions.

### 3.2 Development of variables

The questionnaire from Appendix B asked for the respondents' assessment of the four variables associated with solidarity pressure, namely support for women's football, the fight against racism and discrimination, social commitment and environmental commitment, as shown in Table 5, with responses elicited on a five-point Likert scale.

**Table 5:** Variables related to solidarity pressure.

Variables	Question	Likert scale
Support of women's football	To what extent do you appreciate the fact that the management of a football club supports the development and consolidation of women's football (well-trained staff, strategies to encourage the attendance of fans)?	1. Not at all appreciative 2. Slightly appreciative 3. Moderately appreciative 4. Very appreciative 5. Extremely appreciative
Fight against racism and discrimination	To what extent do you appreciate the fact that the management of a football club supports the fight against racism and all forms of discrimination?	
Social commitment	To what extent do you appreciate the fact that the management of a football club supports social commitment through support programs for foundations, football schools, pet adoptions, etc.?	
Environmental commitment	To what extent do you appreciate the fact that the management of a football club supports pro-environmental activities in the stadium and through the dissemination of activities that contribute to the protection of the environment?	

In addition, a scenario was presented for each variable in which the different sustainability outcomes were inquired about. As shown in Table 6, in the economic dimension, questions were asked about stadium attendance, purchases of merchandise and subscriptions to sports TV channels. Similarly, in the social dimension, questions focused on the impact on reputation and goodwill. In the environmental dimension, the questions focused on environmental practices in the stadium and the development of environmental awareness among fans. As shown in Appendix B, each response option was presented on a Likert scale where (1) strongly disagree, (2) disagree, (3) neither agree nor disagree, (4) agree and (5) strongly agree. In this way, the four solidarity pressure variables are related to the expected sustainability outcomes of football clubs, including economic, social and environmental impacts. Note that the environmental outcomes are only related to the environmental commitment variable, while the economic and social outcomes are related to the four solidarity pressure variables.

**Table 6:** Statements on each variable of solidarity pressure and expected sustainability outcomes.

Pressures regarding solidarity	Statements	Economic outcomes			Social outcomes	Environmental outcomes	
		A. Income from individual and season tickets	B. Income from merchandising	C. Income from sports broadcasting rights	D. Reputation or Goodwill	E. Outcomes of environmental practices in stadiums	F. Development of environmental awareness among fans
Support to women's football	1. If the management of the football club you are a fan of is committed to the development and consolidation of women's football, then:	You would go to the stadium (buy individual and season tickets).	You would buy the football club's merchandise (clothes, caps, jackets, T-shirts, etc.)	You would pay a membership fee to the TV channels that broadcast football matches.	This situation would have a positive effect on the reputation or goodwill of the club.	–	–
Fight against racism and discrimination	2. If the management of the football club you are a fan of is committed to fighting against racism and any form of discrimination, then:	You would go to the stadium (buy individual and season tickets).	You would buy the football club's merchandise (clothes, caps, jackets, T-shirts, etc.)	You would pay a membership fee to the TV channels that broadcast football matches.	This situation would have a positive effect on the reputation or goodwill of the club.	–	–
Social commitment	3. If the management of the football club you are a fan of supports social commitment through is supporting programs for foundations, football schools, pet adoptions, then:	You would go to the stadium (buy individual and season tickets).	You would buy the football club's merchandise (clothes, caps, jackets, T-shirts, etc.)	You would pay a membership fee to the TV channels that broadcast football matches.	This situation would have a positive effect on the reputation or goodwill of the club.	–	–
Environmental commitment	4. If the management of the football club you are a fan of is committed to pro-environmental activities in the stadium and the protection of the environment, then:	You would go to the stadium (buy individual and season tickets).	You would buy the football club's merchandise (clothes, caps, jackets, T-shirts, etc.)	You would pay a membership fee to the TV channels that broadcast football matches.	This situation would have a positive effect on the reputation or goodwill of the club.	This situation would contribute to better environmental care in the stadium	This situation would contribute to the development of environmental awareness

### 3.3 Data collection and analysis

In order to find a representative sample for the study, an infinite population was considered because the population of football fans in Colombia is so large that it cannot be practically counted, and because the population is constantly changing, so there is no fixed total number of people to count. In this sense, the sample size calculation was based on Equation (1), where *Z* is the value in the standard normal distribution associated with the desired confidence level, which in this case is 1.96 for a confidence level of 95%. Also, *p* is the expected proportion in the population, which is assumed to be 0.5 as a conservative value, and *E* is the acceptable margin of error, which in this case is 0.05 for a margin of 5%, resulting in a required sample size of at least 384 surveys.

(1) $$n=\frac{Z^{2}\cdot p\cdot (1-p)}{E^{2}}$$

The recruitment of participants to achieve the sample size was carried out during June–July 2024, using the convenience sampling method, with the aim of obtaining as many responses as possible. In this way, the survey collected responses from 604 individuals, of which 501 responses were validated by obtaining complete and coherent information from the questions asked and by classifying them into one of the four categories of fans identified in the study. The final sample of 501 responses, which also meets the representativeness criteria established for the sample size, was made up of 17.0% direct fans, 34.1% indirect fans, 41.5% passive fans and 7.4% potential fans, i.e., 51.2% active fans and 48.8% non-active fans. The demographic breakdown of the sample shows that 60.3% of respondents are male and 39.7% are female, 58.7% of respondents are aged 18–24, 14.6% are aged 25–34, 12.0% of respondents are aged 35–44 and the remaining 14.8% are aged 45 or older. In addition, 96.0% of respondents live in the region of Antioquia, Colombia, which is home to the football club with the largest number of fans in Colombia and the most recognition in international football club tournaments.

After obtaining the survey responses, and in order to obtain the values of the observable variables of the conceptual model, Table C1 of Appendix C shows how the information from the questionnaire questions was used to calculate values for the variables of solidarity pressure and sustainability outcomes. This allows a structural equation model to be set up and run, whose constructs, as shown in the proposed theoretical model, are solidarity pressure, economic outcomes, social outcomes and environmental outcomes.

Since one of the main contributions of this study is the fact of including the pressures generated by different types of fans, the hypotheses of the conceptual model should be tested in two groups: a model tested with active fans and a model tested with non-active fans. The hypotheses are empirically tested using structural equation modeling, specifically using the partial least squares structural equation modelling (PLS-SEM) method, which is an increasingly applied multivariate analysis technique in management research (93). This allows the analysis of complex relationships between variables, both observed and latent, to test a theoretical framework from a predictive perspective, including a structural path model for formatively measured constructs, without imposing distributional assumptions on the data (94). In addition, we used Smart PLS 4 software since it has an intuitive and easy-to-use interface and offers versatility across a wide range of disciplines, including management and social sciences, with advanced capabilities for multivariate analysis, particularly in PLS-SEM modelling. The software makes it easy to visualize models and obtain results with graphs and has been widely used in Ph.D. theses and advanced research studies and is a suitable choice over older tools such as AMOS and MPLUS.

## 4 Results

The results of this study are organized according to the proposed conceptual model, including both the measurement model and the structural model, as recommended by Marrucci et al. (95). The structural model includes the results of hypothesis testing for the active fan model and the results for the non-active fan model.

### 4.1 Measurement model

The path coefficients are useful to test whether the predictor variables contribute to explaining the variance of the endogenous variable (96). These are significant if they are greater than 0.2 (97). However, to be more stringent, they should be greater than 0.3 (98).

To check the reliability of the constructs, the Cronbach Alpha analysis is required, which, according to Nunnally (99), should be greater than 0.7. Similarly, to check the internal consistency of the model, an analysis of the Average Variance Extracted (AVE) should be carried out, which is only applicable to reflective indicators, which is the case in this research. According to Chin (100), this value should be greater than 0.5. Similarly, the structured evaluation of the model must be verified by the R-squared, which according to Miles (101) should be greater than 0.1.

To analyze the individual reliability of the constructs, outer loadings should be checked, which should be greater than 0.707 according to Carmines and Zeller (102) or 0.55 according to Hwang et al. (103). In this work, for the outer loadings to be significant, we take as a criterion that the indicators must be higher than 0.707, as this is the one used as a reference in the Smart PLS 4 software. Multicollinearity analysis (variance inflation factor—VIF) helps to understand whether some variables explain the same construct as other variables. For some authors, a high and unacceptable value of VIF would be equal to ten, and a low and therefore acceptable value would be less than four (104). Other authors argue that this value should not be higher than 5 (105) and still others argue that this value should not be higher than three (95). By default, Smart PLS 4 assumes multicollinearity problems with a VIF greater than five.

### 4.2 Structural model

#### 4.2.1 Results of the model based on active fans

**Figure 2:**
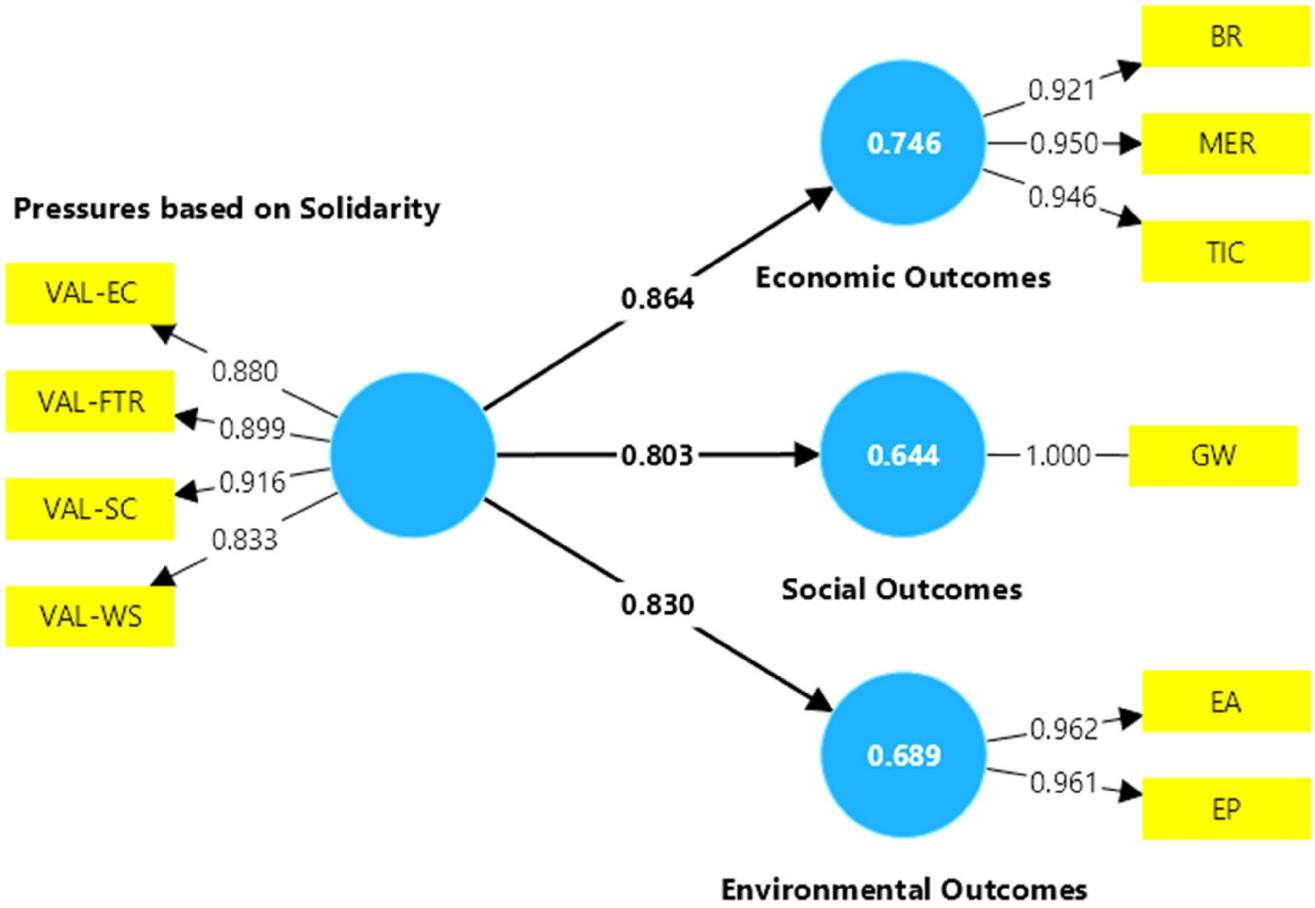
Model results for active fans.

The results of the model that includes active fans are presented in Figure 2. Since all path coefficients are greater than 0.3, the model of the active fans supports the hypothesis that solidarity pressures have a significant impact on the economic, social and environmental outcomes of football clubs. While all hypotheses are significant, for active fans the greatest impact of their solidarity pressure would be on economic performance.

Table 7 shows that for each sustainability outcome Cronbach's Alpha values are greater than 0.7 and the AVEs are greater than 0.5, therefore the constructs are reliable and the AVEs indicate that the model has internal consistency. Since all coefficients are greater than 0.707, it is concluded that the indicators are reliable. Likewise for all sustainability outcomes, the R-squared and adjusted R-squared are greater than 0.1, so the model is considered to have an adequate structural fit.

**Table 7:** Main results of the model constructs for active fans.

Constructs	Path coefficients	*R*-square	*R*-square adjusted	Cronbach's alpha	Average variance extracted (AVE)
Economic Outcomes	0.864	0.746	0.745	0.933	0.881
Environmental Outcomes	0.830	0.689	0.688	0.918	0.924
Social Outcomes	0.830	0.644	0.643	0.905	0.779

According to Table 8, for solidarity-based pressures, the most highly rated indicators were goodwill (GW), environmental awareness (EA), and Environmental practices at stadiums (EP). In terms of economic results, the indicators MER and TIC received the highest rating. On the other hand, among the solidarity-based pressures for active fans, the variable VAL-SC, representing social commitment from football clubs, was the most highly rated, followed by fight against racism and discrimination VAL-FTR.

**Table 8:** Outer loadings and variance inflation factors for active fans.

Variables	Outer loadings	VIF
BR	0.921	3.156
EA	0.962	3.579
EP	0.961	3.579
GW	1.000	1.000
MER	0.950	4.900
TIC	0.946	4.467
VAL-EC	0.880	2.625
VAL-FTR	0.899	3.077
VAL-SC	0.916	3.466
VAL-WS	0.833	2.211

Table 8 shows that the model with active fans does not present problems of collinearity, but the variable MER would be very close to collinearity. In this sense, the variables that best explain the economic results are TIC and BR. Part of this explanation would be related to the fact that active fans are made up of direct and indirect fans, and that direct fans value going to the stadium more, while indirect fans value following the club through broadcasting channels. Thus, both the outer loadings analysis and the multicollinearity analysis allow us to establish that the observable variables of the model are reliable and explain independently the constructs to which they belong.

#### 4.2.2 Results of the model based on non-active fans

**Figure 3:**
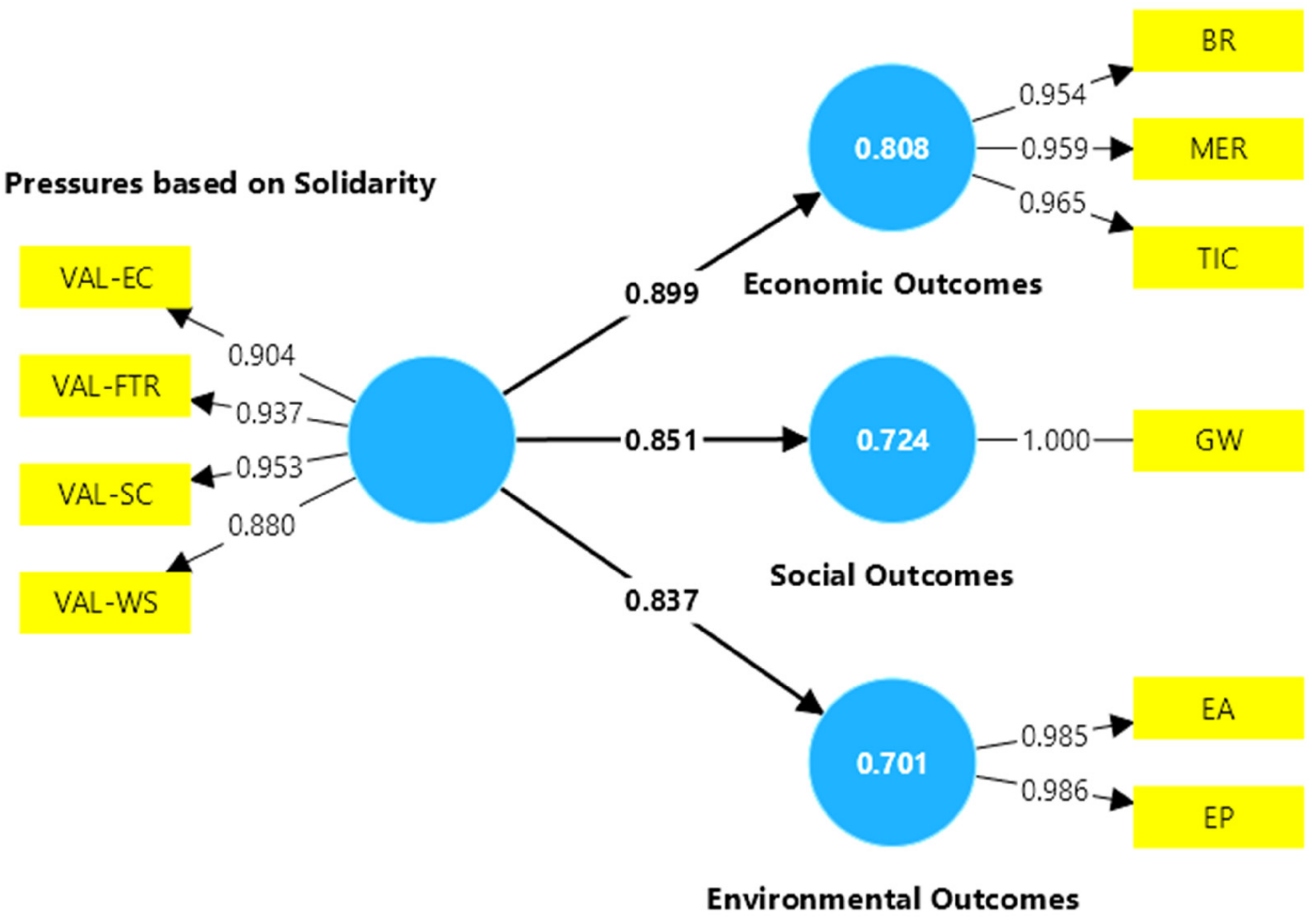
Model results for non-active fans.

Figure 3 shows the results of the model that includes non-active fans, where it is noted that all path coefficients are greater than 0.3, so the model for non-active fans supports the hypotheses that solidarity pressure has a significant impact on the economic, social and environmental performance of football clubs. Based on the results in Table 9, the greatest impact of solidarity pressure from non-active fans would be on economic performance. On the other hand, the VAL-SC indicator, which represents social commitment, was the most valued among the solidarity pressures for non-active fans, followed by the fight against racism (VAL-FTR), as was the case for active fans.

**Table 9:** Main results of the model constructs for non-active fans.

Constructs	Path coefficients	*R*-square	*R*-square adjusted	Cronbach's alpha	Average variance extracted (AVE)
Economic Outcomes	0.899	0.808	0.808	0.957	0.921
Environmental Outcomes	0.837	0.701	0.699	0.970	0.971
Social Outcomes	0.851	0.724	0.723	0.938	0.845

Furthermore, Table 9 shows that both Cronbanch's alphas are higher than 0.7 and the AVEs are higher than 0.5, so the constructs are reliable, and the model has internal consistency for the case of non-active fans. Furthermore, for the economic, social and environmental outcomes, the R-squared and adjusted R-squared are greater than 0.1, so the model is considered to have an adequate structural fit.

Table 10 shows that all the outer loadings coefficients are greater than 0.707, which indicates that the indicators of the model for inactive fans are reliable. However, in terms of economic outcomes, the ICT, MER and BR variables show a high collinearity between them according to the VIF, so the two variables with the highest VIF are eliminated. In the environmental outcomes, both EP and EA have the same collinearity value, indicating that they explain similar aspects in the model, so that statistically any of them could be excluded. In this case, the EA indicator was excluded because it is considered that there must be a diffusion of environmental practices prior to the generation of environmental awareness, and it has a lower outer loading coefficient value than EP. In addition, in order to develop environmental awareness, a process of sensitization of the fans must first be generated (106). Regarding the variables related to solidarity pressures, the VIF values indicate that the VAL-SC indicator, which represents social commitment, has a higher collinearity value, so it was decided to eliminate it from the model, as other variables can explain similar relationships in the model.

**Table 10:** Outer loadings and variance inflation factors for non-active fans.

Variables	Outer Loadings	VIF
BR	0.954	5.214
EA	0.985	8.770
EP	0.986	8.770
GW	1.000	1.000
MER	0.959	5.873
TIC	0.965	6.436
VAL-EC	0.904	3.277
VAL-FTR	0.937	5.003
VAL-SC	0.953	6.177
VAL-WS	0.880	2.947

Once the variables with high collinearity have been eliminated, the structural equation model is re-run, resulting in the VIF values shown in Table 11. This new model better represents the valuation of non-active fans by solving collinearity problems, considering broadcasting rights (BR) for economic performance, goodwill (GW) for social performance and environmental practices (EP) for environmental performance, and considering solidarity pressures related to environmental commitment, the fight against racism and discrimination and women's football.

**Table 11:** Multicollinearity with VIF correction.

Collinearity statistics (VIF)	VIF
BR	1.000
EP	1.000
GW	1.000
VAL-EC	2.734
VAL-FTR	3.622
VAL-WS	2.639

For both models, considering active and non-active fans, all hypotheses were found to be significant, with H1 being the most valued, which relates solidarity pressure to economic performance. Similarly, for both models, all indicators were reliable, with fight against racism and discrimination (FTR) being the most valued. In terms of economic outcomes, income from individual and season tickets (ICT) is the most relevant indicator for active fans, while income from broadcasting rights is the most relevant for non-active fans. For social outcomes, both models agree that goodwill (GW) is a reliable indicator. Finally, for environmental outcomes, environmental awareness (EA) is the most relevant indicator for active fans and environmental practices (EP) is the most relevant for non-active fans.

## 5 Discussions

Regarding the results of the study, all hypotheses were significant in the two models, but the one that was most valued by both types of fans was the economic outcome. This could be explained by the fact that the survey is not based on decisions or objective data, but on fans' evaluations, which may not be the case in practice. In other words, the decision to buy single or season tickets, merchandise and even broadcasting rights may not actually be made. In this respect, the two indicators that stood out in the active fan model were single and season tickets, and income from broadcasting rights. The explanation for this is that this group is made up of direct fans, who place a high value on being in the stadium, and indirect fans, who visit the stadium occasionally or follow their football clubs through TV broadcast.

In the active fans model, the best indicators of solidarity pressure were the social commitment of the football clubs (VAL-SC) and the fight against racism and discrimination (VAL-FTR). In the first case, the explanation could be related to the fact that most football clubs in Colombia already have consolidated social programs through which fans perceive their social commitment. These programs include working with communities, supporting children's foundations and programs with vulnerable populations, and promoting pet adoption plans. In the second case, the fight against racism and discrimination is a program promoted by FIFA and highly valued by fans in Colombia. Similarly, the model of non-active fans reaches a consensus that the most relevant indicator is the fight against racism (VAL-FTR).

On the other hand, in the model of non-active fans, the most valued indicator was the income from broadcasting rights, which is fully consistent with this typology, since neither passive fans nor potential fans usually attend the stadium or buy the goods and services offered by the football club. Therefore, their main means of following the sport is through broadcasting channels or virtual media. In this regard, the latter should be an aspect on which the managers of football clubs should focus, since in European leagues a good part of the income comes from these sources, while in the Colombian league the income related to this concept is still low (43). The above indicates that, several stakeholders such as football institutions (22), which in the case of Colombia would be the Colombian Football Major Division (Dimayor) and the Colombian Football Federation; market and social institutions (21), such as the subscription broadcasters (107) and the managers of the football clubs (27), must work together to ensure that the business model derived from the broadcasting rights is beneficial to the parties involved, as this translates into an improvement in the financial sustainability of the football clubs.

In terms of social outcomes, both models agree on the very high valuation of the goodwill of the football clubs. This goodwill is given by the recognition of the orientation towards programs such as the support of women's football (57, 58, 76, 77), the fight against racism and discrimination (80, 81), and the promotion of the environment (5, 23, 36). In terms of environmental outcomes, both models positively evaluate the efforts made by football clubs in terms of environmental practices, as well as their commitment to raising environmental awareness among fans. In fact, these two variables have a very similar behavior, with a slightly better performance of the indicator of environmental practices (EP) for non-active fans and the development of environmental awareness (EA) for active fans.

The theoretical model that underpins the measurement can be extended to other countries and regions, including Europe, because solidarity pressures have the same conceptual basis (69), and the issues of social engagement, the fight against racism, the promotion of women's football and climate change are part of the SDGs agenda adopted by football institutions such as FIFA, CONMEBOL or UEFA. In addition, the sources of income of most football clubs are common: income from broadcasting rights, income from the sale of individual and season tickets, and merchandising of the football club. Similarly, in the social dimension, works carried out in European leagues show that the most common sustainability outcome is goodwill (20–22), while environmental practices and the development of environmental awareness as the most common indicators in these studies (11, 23, 36).

Applying the model in Europe could change the results, since aspects such as the percentage of fans type, football governance and the business models of European football clubs could differ significantly. In Germany, for example, there is the 50 + 1 rule, which means that part of the ownership of football clubs belongs to the fans, while Colombian professional football clubs are private organizations. Similarly, environmental commitment is more established in European leagues than in South America, including Colombia, which explains why European leagues are at a stage of developing environmental awareness among fans, while the Colombian league is just beginning to disseminate environmental practices in the stadium. In this sense, social commitment in Colombia is more consolidated than environmental commitment, as Colombian football clubs have a good development of social programs, while environmental plans, programs and activities are still incipient. The results of this study support this assertion.

### 5.1 Theoretical implications

This paper aims to advance the state of the art of fan pressure, as it differs from previous works by presenting some types of pressure generated by fans, known as solidarity-based pressure (69), from which four variables have been identified: the empowerment of women's football (VAL-WS) (57, 77), the fight against racism and discrimination (VAL-FTR) (78, 80, 81), social commitment reflected in social programs with the community (VAL-SC) (32), and environmental commitment, which has two components, environmental practices in stadiums (VAL-EP) (20–22, 106) and diffusion of environmental awareness among fans (23, 36).

Similarly, another differentiating factor presented in this study is the focus on sustainability performance based on the triple bottom line of economic, social and environmental dimensions. Thus, economic outcomes were related to income from single and season ticket sales (TIC) (30, 47), merchandising of the football club (MER) (30, 32) and income from broadcasting rights (BR and income from broadcasting rights (BR). In the case of social outcomes, aspects such as goodwill and reputation (GW), developed in works such as Daddi et al. (20), Daddi et al. (21), and Todaro et al. (22), have been included. Environmental outcomes are associated either with environmental practices in the stadium (EP) or with the club's commitment to generating environmental awareness among fans (EA) (11, 23, 36, 106). All these elements constitute the novelty of this study.

Another contribution of this study is that it addresses a theoretical model with two types of fans, considered as active fans and non-active fans. Active fans can be direct or indirect (29), where direct fans are those who continuously attend the stadium by purchasing individual tickets or season ticket plans (51, 52), while indirect fans sometimes attend and follow their clubs through television channels, many of them with subscription payments (107, 108). For their part, non-active fans can be passive or potential. Passive ones would be those who, as fans, have a very low level of loyalty (26, 45), while potential ones are those generally known as green consumers (11, 23), who are motivated by issues related to sustainability, climate change or the SDG agenda. Although fan typologies are not new, what is innovative is the study of how these typologies generate different pressures on football club managers, as the pressures generated by fans differ according to their level of loyalty and support for the football club (43).

### 5.2 Practical implications

In the case of Colombian football, the main revenues for football clubs come mainly from broadcasting and television rights, the sale of individual and season tickets, and the sale of merchandise (42). Regarding broadcasting rights, there is a great potential for revenue generation, where football institutions such as the Colombian Football Association (Dimayor) and the Colombian Football Federation, subscription broadcasters and football club managers need to work together so that the business model derived from broadcasting rights is beneficial to the parties involved, as this translates into an improvement in their financial sustainability.

The conventional approach to the management of football clubs has focused on the pursuit of improved sporting performance in order to enhance reputation (43). The results of this study show that all solidarity pressures, including social engagement, anti-racism, promotion of women's football and environmental engagement, have a positive impact on the reputation or goodwill of football clubs, which in turn can generate positive societal impacts. These include supporting children's foundations (74), combating racism and discrimination (82), and promoting women's football (57). In addition to contributing to a fairer society, these actions can also contribute to sporting performance and the valorization of the football club (92).

Similarly, the results related to the environmental dimension offer valuable insights for the management of football clubs. These insights include the potential for environmental practices to be applied in stadiums and the possibility of a formative component linked to the intention of increasing fans' environmental awareness. Although the project is still in its infancy, the recommended next step is to disseminate environmental practices and raise awareness among fans (106). This does not mean that football clubs have a duty to replace the work of governmental institutions such as national, regional and local governments (22), but rather that they should complement them, using the potential for social change that football clubs have through their fan base (36).

Due to the affective connection of fans, it might be easier to retain active fans rather than constantly finding ways to engage new fans because active fans are almost unconditional fans for life (45, 53). Furthermore, the pressures associated with solidarity, which are linked to social pressures (19), such as gender equality promoted by the 2030 Agenda and reflected in the empowerment of women's football, environmental protection promoted by the Environmental Pledge, represent valuable opportunities for football clubs to increase their fan base through activities aimed at attracting passive and potential fans (43).

However, these activities should provide new experiences for both active and non-active fans. In this sense, potential fans can be encouraged to attend women's football matches that include a cultural or artistic experience, complemented by concerts and incentives that involve the participation of new fans. Similarly, events can be organized to promote environmental practices and the development of environmental awareness through participatory activities that reward fans. In this sense, the management of football clubs will focus not only on football matches, but also on related goods and services through an experience economy. This would tap into the emotions of fans through a sophisticated value proposition focused on the fan experience (24).

## 6 Conclusions

This research responds to the field of stakeholder pressure and its impact on organizational sustainability performance, which suggests that football clubs implement sustainability goals, strategies, practices and policies not because of philanthropy, but because of stakeholder pressure. In the sports sector, particularly football clubs, the governing bodies of these organizations seem to act in a sustainable manner, mainly due to pressure from various stakeholders. This approach focuses on one of the most important stakeholders in the football sector, the fans, because they represent stakeholders that are different from others due to the existence of the “affective connection of the fans”, which generates feelings of loyalty and a sense of urgency on the part of the fans, which can translate into benefits and returns in the economic, social and environmental dimensions.

This article proposes a novel methodology because it looks at different types of fans, recognizing that each type of fan may be interested in generating different pressures, and it extends the outcomes of sustainability such as income from ticket sales, merchandising, broadcasting rights (economic outcomes); the impact on goodwill and reputation (social outcomes); and the consideration of both environmental practices and the development of environmental awareness (environmental outcomes).

In terms of solidarity pressures, although all of them are important, the one most valued by fans was social commitment, followed by the fight against racism, the promotion of women's football and the fight against racism. This result is understandable, since Colombian football clubs have already consolidated the development of social program and activities in favor of different types of vulnerable communities, and the strengthening of the fight against racism and women's football are part of international agendas, such as the SDGs, which find a response in sports institutions, such as FIFA and CONMEBOL, as well as in government institutions at different levels, such as national and local governments. All this has led to their acceptance by different types of fans.

On the other hand, the variable that belongs to the solidarity pressures with the least relevance, although it was rated well, was the environmental commitment (EC). Although it is also part of important international agendas, such as the SDGs and the Paris Agreement, professional football clubs in Colombia pay less attention to this aspect than others, so such practices are minimal. The first step is to raise awareness to help fans extend their environmental actions and practices beyond the stadium to their workplaces, homes and communities. In this sense, football organizations could be an important ally for governments in meeting their climate commitments and achieving some of the SDGs.

### 6.1 Limitations and future work

The model proposed in this study has some limitations. The first is the sample size of the different types of fans, especially direct fans, who represent 33% of active fans and only 17% of the total sample, and the participation of potential fans, who represent 15% of non-active fans and only 7% of the total sample. This situation makes the ratings of indirect and passive fans (the typologies with the least emotional attachment) weigh more heavily in the proposed model. The second limitation is related to the collinearity generated by some variables related to solidarity pressures in terms of social engagement, such as women's football and the fight against racism and discrimination. This could be corrected by including the variables of women's football and the fight against racism in the variables explaining social engagement, as these activities are also related to the social engagement of football clubs. Thirdly, the impact of the evaluations may be influenced by the percentage of fans responding to the survey who are fans of a particular football club and by the sporting performance of that club at the time of the survey. For the latter, a sample of fans of different Colombian football club was sought. These limitations will have to be considered in future work that seeks to replicate the proposed model in different South American countries. Despite these limitations, the results are consistent and reveal important aspects on which football club managers could focus their management.

Future research into the pressure of different fan typologies on sustainability outcomes could be conducted on a larger scale and with larger samples in the same country, and similar studies could be replicated in countries other than European leagues, such as the major leagues in South and Central America. This would allow comparisons to be made based on fan pressure and whether it has a significant impact on sustainability outcomes. Similarly, future studies could compare the research findings on fan pressure with the attitudes and actions of managers. Similarly, future research could include a wider range of stakeholders in the football sector and their impact on sustainability performance.

## Data Availability

The raw data supporting the conclusions of this article will be made available by the authors, without undue reservation.

## Appendix A

**Table A1:** Variables for the conceptual model of solidarity-based pressures.

Constructs/latent variables	Observed variables	Abbreviation	Calculation method
Pressures based on solidarity	Fans' valuation of women's football.	VAL-WS	Response to support for women's football on a Likert scale of 1–5 (B.1.1)
	Fans' valuation of the fight against racism	VAL-FTR	Response to support for fight against racism and discrimination on a Likert scale of 1–5 (B.1.2)
	Fans' valuation towards the social commitment of the football club.	VAL-SC	Response to support for social commitment on a Likert scale of 1–5 (B.1.3)
	Fans' valuation of the football club's commitment to the environment.	VAL-EC	Response to support for environmental commitment on a Likert scale of 1–5 (B.1.4)
Economic Outcomes	Income from single and season tickets	TIC	Represents the average of the four questions on income from single and seasons tickets (B.2.1, B.3.1, B.4.1, B.5.1)
	Income from merchandising	MER	Represents the average of the four questions on merchandising income (B.2.2, B.3.2, B.4.2, B.5.2)
	Income from sports broadcasting rights	BR	Represents the average of the 4 questions on broadcasting rights income (B.2.3, B.3.3, B.4.3, B.5.3)
Social Outcomes	Reputation or goodwill	GW	Represents the average of the four questions on goodwill (B.2.4, B.3.4, B.4.4, B.5.4)
Environmental Outcomes	Environmental practices at stadiums	EP	Response to support for commitment to environmental practices at the stadium on a Likert scale of 1–5 (B.5.5)
	Development of environmental awareness among fans	EA	Response to support for development of environmental awareness among fans on a Likert scale of 1–5 (B.5.6)

## Appendix B

Perception survey on fan solidarity pressure and its impact on the sustainability of football clubs.

The following survey aims to analyze the influence of fan pressure on the sustainability performance of football clubs, covering economic, social and environmental dimensions. The idea is that companies, such as football clubs, decide to work on sustainability issues when they feel pressure from stakeholders. Fans are one of the most important stakeholders. This is because the moral owners of football clubs can be seen as the fans, as they have a strong emotional/affective attachment to the clubs, which legitimizes and commits them. Because of this affective connection, football clubs have an important potential to generate changes in society.

This questionnaire will be used for academic purposes, and the answers generated could provide elements to improve fan management in football clubs in Colombia. In this sense, the data you provide will not be used for other purposes and will not, under any circumstances, reveal personal or sensitive data of those who respond to the questionnaire, as proposed in the Personal Data Protection Act or Law 1581 of 2012.

### Part A: demographic profile and types of fans

**Table B1:** Respondents' demographic profile.

Code	Question	Response options
A.1	First and last names	Open response
A.2	Department/State of residence	Selection of one of Colombia's 32 departments
A.3	Municipality of residence	Open response
A.4	What is your age range?	Under 18 years of age
		18 years to 24 years
		25 years to 34 years
		35 years to 44 years
		45 years to 54 years
		Over 54 years of age
A.5	What is the gender with which you best identify?	Male
		Female
		Other, which one?

**Table B2:** Fan types.

Code	Question	Response options
A.6	Which of the following statements best describes the type of fan you are?	• You visit the stadium on a regular basis with an individual or season ticket. • You go to the stadium sporadically (for important matches or finals) or follow your football club regularly via a TV channel that broadcasts the matches (ESPN, Sky Sports, GO Sports, Eurosport, Win Sport). • You support a football club, you're happy when they do well, but you don't go to the stadium or follow the club via a TV channel that broadcasts the matches. • You are not a fan of a particular club, but if a football club is involved in activities that promote women's football, environmental and social practices, you might consider supporting them directly or indirectly.

### Part B: solidarity pressures and their relation to dimensions of sustainability

**Table B3:** Solidarity pressures.

Code	Question	Response options
B.1.1	To what extent do you appreciate the fact that the management of a football club supports the development and consolidation of women's football (well-trained staff, strategies to encourage the attendance of fans)?	1. Not at all appreciative 2. Slightly appreciative 3. Moderately appreciative 4. Very appreciative 5. Extremely appreciative
B.1.2	To what extent do you appreciate the fact that the management of a football club supports the fight against racism and all forms of discrimination?	1. Not at all appreciative 2. Slightly appreciative 3. Moderately appreciative 4. Very appreciative 5. Extremely appreciative
B.1.3	To what extent do you appreciate the fact that the management of a football club supports social commitment through support programs for foundations, football schools, pet adoptions, etc.?	1. Not at all appreciative 2. Slightly appreciative 3. Moderately appreciative 4. Very appreciative 5. Extremely appreciative
B.1.4	To what extent do you appreciate the fact that the management of a football club supports pro-environmental activities in the stadium and through the dissemination of activities that contribute to the protection of the environment?	1. Not at all appreciative 2. Slightly appreciative 3. Moderately appreciative 4. Very appreciative 5. Extremely appreciative

**Table B4:** Support for women's football.

Code	Question	Response options
B.2.1	If the management of the football club you are a fan of is committed to the development and consolidation of women's football, then you would go to the stadium (buy individual and season tickets).	1. Strongly disagree 2. Disagree 3. Neither agree nor disagree 4. Agree 5. Strongly agree
B.2.2	If the management of the football club you are a fan of is committed to the development and consolidation of women's football, then you would buy the football club's merchandise (clothes, caps, jackets, T-shirts, etc.).	1. Strongly disagree 2. Disagree 3. Neither agree nor disagree 4. Agree 5. Strongly agree
B.2.3	If the management of the football club you are a fan of is committed to the development and consolidation of women's football, then you would pay a membership fee to the TV channels that broadcast football matches.	1. Strongly disagree 2. Disagree 3. Neither agree nor disagree 4. Agree 5. Strongly agree
B.2.4	If the management of the football club you are a fan of is committed to the development and consolidation of women's football, then this situation would have a positive effect on the reputation or goodwill of the club.	1. Strongly disagree 2. Disagree 3. Neither agree nor disagree 4. Agree 5. Strongly agree

**Table B5:** Fight against racism and discrimination.

Code	Question	Response options
B.3.1	If the management of the football club you are a fan of is committed to fighting against racism and any form of discrimination, then you would go to the stadium (buy individual and season tickets).	1. Strongly disagree 2. Disagree 3. Neither agree nor disagree 4. Agree 5. Strongly agree
B.3.2	If the management of the football club you are a fan of is committed to fighting against racism and any form of discrimination, then you would buy the football club's merchandise (clothes, caps, jackets, T-shirts, etc.).	1. Strongly disagree 2. Disagree 3. Neither agree nor disagree 4. Agree 5. Strongly agree
B.3.3	If the management of the football club you are a fan of is committed to fighting against racism and any form of discrimination, then you would pay a membership fee to the TV channels that broadcast football matches.	1. Strongly disagree 2. Disagree 3. Neither agree nor disagree 4. Agree 5. Strongly agree
B.3.4	If the management of the football club you are a fan of is committed to fighting against racism and any form of discrimination, then this situation would have a positive effect on the reputation or goodwill of the club.	1. Strongly disagree 2. Disagree 3. Neither agree nor disagree 4. Agree 5. Strongly agree

**Table B6:** Social commitment.

Code	Question	Response options
B.4.1	If the management of the football club you are a fan of supports social commitment through support programs for foundations, football schools, pet adoptions, then you would go to the stadium (buy individual and season tickets).	1. Strongly disagree 2. Disagree 3. Neither agree nor disagree 4. Agree 5. Strongly agree
B.4.2	If the management of the football club you are a fan of supports social commitment through support programs for foundations, football schools, pet adoptions, then you would buy the football club's merchandise (clothes, caps, jackets, T-shirts, etc.).	1. Strongly disagree 2. Disagree 3. Neither agree nor disagree 4. Agree 5. Strongly agree
B.4.3	If the management of the football club you are a fan of is supporting social commitment through support programs for foundations, football schools, pet adoptions, then you would pay a membership fee to the TV channels that broadcast football matches.	1. Strongly disagree 2. Disagree 3. Neither agree nor disagree 4. Agree 5. Strongly agree
B.4.4	If the management of the football club you are a fan of supports social commitment through support programs for foundations, football schools, pet adoptions, then this situation would have a positive effect on the reputation or goodwill of the club.	1. Strongly disagree 2. Disagree 3. Neither agree nor disagree 4. Agree 5. Strongly agree

**Table B7:** Environmental commitment.

Code	Question	Response options
B.5.1	If the management of the football club you are a fan of is committed to pro-environmental activities in the stadium and the protection of the environment, then you would go to the stadium (buy individual and season tickets).	1. Strongly disagree 2. Disagree 3. Neither agree nor disagree 4. Agree 5. Strongly agree
B.5.2	If the management of the football club you are a fan of is committed to pro-environmental activities in the stadium and the protection of the environment, then you would buy the football club's merchandise (clothes, caps, jackets, T-shirts, etc.).	1. Strongly disagree 2. Disagree 3. Neither agree nor disagree 4. Agree 5. Strongly agree
B.5.3	If the management of the football club you are a fan of is committed to pro-environmental activities in the stadium and the protection of the environment, then you would pay a membership fee to the TV channels that broadcast football matches.	1. Strongly disagree 2. Disagree 3. Neither agree nor disagree 4. Agree 5. Strongly agree
B.5.4	If the management of the football club you are a fan of is committed to pro-environmental activities in the stadium and the protection of the environment, then this situation would have a positive effect on the reputation or goodwill of the club.	1. Strongly disagree 2. Disagree 3. Neither agree nor disagree 4. Agree 5. Strongly agree
B.5.5	If the management of the football club you are a fan of is committed to pro-environmental activities in the stadium and the protection of the environment, then this situation would contribute to better environmental care in the stadium.	1. Strongly disagree 2. Disagree 3. Neither agree nor disagree 4. Agree 5. Strongly agree
B.5.6	If the management of the football club you are a fan of is committed to pro-environmental activities in the stadium and the protection of the environment, then this situation would contribute to the development of environmental awareness.	1. Strongly disagree 2. Disagree 3. Neither agree nor disagree 4. Agree 5. Strongly agree

## Appendix C

**Table C1:** Calculation method for the variables for the conceptual model.

Abbreviation	Calculation method
VAL-WS	Response to support for women's football on a Likert scale of 1–5 (B.1.1)
VAL-FTR	Response to support for fight against racism and discrimination on a Likert scale of 1–5 (B.1.2)
VAL-SC	Response to support for social commitment on a Likert scale of 1–5 (B.1.3)
VAL-EC	Response to support for environmental commitment on a Likert scale of 1–5 (B.1.4)
TIC	Represents the average of the four questions on income from single and seasons tickets (B.2.1, B.3.1, B.4.1, B.5.1)
MER	Represents the average of the four questions on merchandising income (B.2.2, B.3.2, B.4.2, B.5.2)
BR	Represents the average of the 4 questions on broadcasting rights income (B.2.3, B.3.3, B.4.3, B.5.3)
GW	Represents the average of the four questions on goodwill (B.2.4, B.3.4, B.4.4, B.5.4)
EP	Response to support for commitment to environmental practices at the stadium on a Likert scale of 1–5 (B.5.5)
EA	Response to support for development of environmental awareness among fans on a Likert scale of 1–5 (B.5.6)
